# The use of real-world evidence to generate cost analysis of antibiotic susceptibility testing (AST) in patients with *Helicobacter pylori* treatment failure in Thailand: A large population-based study

**DOI:** 10.1016/j.heliyon.2024.e39189

**Published:** 2024-10-10

**Authors:** Natsuda Aumpan, Pornpen Gamnarai, Arti Wongcha-Um, Muhammad Miftahussurur, Yoshio Yamaoka, Ratha-korn Vilaichone

**Affiliations:** a Center of Excellence in Digestive Diseases and Gastroenterology Unit, Department of Medicine, Thammasat University, Pathumthani, Thailand; b Department of Medicine, Chulabhorn International College of Medicine (CICM) at Thammasat University, Pathumthani, Thailand; c Department of Biochemistry, Faculty of Medicine, Thammasat University, Pathumthani, Thailand; d Division of Gastroentero-Hepatology, Department of Internal Medicine, Faculty of Medicine, Universitas Airlangga, Surabaya, Indonesia; e Department of Medicine, Michael E. DeBakey VA Medical Center and Baylor College of Medicine, 2002 Holcombe Boulevard, Houston, TX, 77030, USA; f Department of Environmental and Preventive Medicine, Oita University Faculty of Medicine, Yufu, Japan; g Research Center for Global and Local Infectious Diseases, Oita University, Yufu, Japan

**Keywords:** *Helicobacter pylori*, Treatment failure, Antibiotic resistance, Cost analysis

## Abstract

**Background:**

*H. pylori* eradication is effective for gastric cancer prevention. Treatment failure is caused by increased antibiotic resistance. This study aimed to determine eradication rates and perform cost analysis between susceptibility-guided therapy and empirical treatment in patients with *H. pylori* treatment failure.

**Methods:**

This retrospective cohort study included patients with dyspepsia undergoing gastroscopy at tertiary care center in Thailand from March 2014 to October 2021. Treatment failure was defined as persistent *H. pylori* infection after ≥1 regimen completion. Early AST was defined as AST performed shortly after first-line treatment failure. Demographic data, AST results, eradication regimens, and medication costs were collected from database and reviewed.

**Results:**

Of 1080 patients with *H. pylori* infection, 315 had treatment failure (mean age 58.4 years; 44.4 % males). AST of 85 strains demonstrated resistance to levofloxacin (57.6 %), metronidazole (51.8 %), clarithromycin (44.7 %), and amoxicillin (4.7 %). In multivariate analysis, sequential therapy was significantly associated with treatment failure (OR 1.66; 95%CI 1.01–2.74, p = 0.045), whereas vonoprazan-containing therapy was related to treatment success (OR 1.60; 95%CI 1.04–2.48, p = 0.034). Medication nonadherence (OR 37.97; 95%CI 8.97–160.65, p < 0.001) contributed to treatment failure. Susceptibility-guided therapy provided better eradication rate than empirical therapy (97.5% vs. 65.5 %, OR 20.54; 95%CI 4.92-85.81, p < 0.001) in treatment failure group. Twenty-four patients had early AST, while 61 had AST after treatment failures. Most patients with early AST achieved treatment success by second-line eradication. Early AST provided significantly lower total average cost of treatment than group without AST ($368.2 vs. $402.0 per patient, p = 0.034) and AST after treatment failures ($368.2 vs. $752.8 per patient, p < 0.001). Early AST group had the lowest cost of subsequent medication, posttreatment urea breath test, and hospital visits.

**Conclusion:**

Susceptibility-guided therapy provided significantly higher eradication rate than empirical therapy in patients with treatment failure. Early AST might be a cost-effective strategy for *H. pylori* eradication after failed therapy and can prevent unnecessary antibiotic use in Thailand.

## 1 Introduction

*Helicobacter pylori (H. pylori)*, a Gram-negative, rod-shaped bacterium, regularly causes chronic gastric mucosal inflammation. *H. pylori* infection results in a wide range of diseases from asymptomatic chronic gastritis, peptic ulcer disease, to gastric cancer [1]. Over 4 billion people worldwide had *H. pylori* infection [2]. The prevalence of *H. pylori* infection varied among countries from 18.9 % to 87.7 % [2]. Approximately one third of Thai population were infected with *H. pylori* as reported in the nationwide study [3]. Early diagnosis and eradication of *H. pylori* infection are recommended to reduce risk of gastric cancer [4,5]. Treatment goal for *H. pylori* infection is achieving >90 % eradication rate [6]. However, the cure rate of standard triple therapy as first-line regimen in Thailand has been decreasing to 80 % [7]. One of the most important barriers to treatment success is rising antibiotic resistance [8]. As clarithromycin is frequently included in the first-line regimen composition, its resistance has a significant impact on eradication success. Therefore, susceptibility test is important to determine regional antibiotic resistance patterns to develop the most effective therapy in an area with high levels of antibiotic resistance [4].

Antimicrobial susceptibility testing (AST) is an in vitro test to determine activity of antibiotics that can effectively inhibit the growth of bacteria. The previous recommendation stated that *H. pylori* culture and AST should be performed after treatment failure [9], while the current guideline recommends that AST should be routinely done even before first-line treatment to prevent antibiotic overuse and promote antibiotic stewardship [4]. However, several issues have to be considered when performing culture and AST including test availability, proper specimen handling, microbiological expertise, and costs. Prior studies evaluating cost-effectiveness between susceptibility-guided treatment and empirical therapy demonstrated inconsistent results. Although these studies reported significantly higher eradication rates in tailored treatment group than empirical treatment group for first-line eradication [[10], [11], [12]], tailored treatment was cost-effective in two studies [11,12]. For refractory *H. pylori* infection, one previous study reported higher cost when using genotypic resistance-guided therapy compared to empirical therapy without difference in eradication rates [13].

Nowadays, a rising number of patients experience *H. pylori* treatment failure because of increasing antibiotic resistance. AST is recommended after treatment failure [7]. Until now, there have been insufficient data regarding eradication rates of therapies after treatment failure in Thailand. This study aimed to determine eradication rates of susceptibility-guided therapy and empirical treatment in patients with refractory *H. pylori* infection in Thailand. Moreover, this study also performed cost analysis between AST-guided therapy and empirical treatment.

## 2 Methods

### 2.1 Study design

This was a retrospective cohort study conducted at tertiary care center in Thailand. Retrospective chart review between March 6, 2014, and October 31, 2021 was performed. This study included patients aged >15 years with refractory *H. pylori* infection defined as positive rapid urease test, histopathology, or culture after completing one or more eradication regimen(s). Patients’ demographic data, comorbidities, endoscopic results, *H. pylori* culture and AST, eradication regimens, and treatment cost were extracted from the medical database and extensively reviewed. *H. pylori* treatment at each follow-up visit was recorded. The exclusion criteria were patients who did not have confirmatory testing for *H. pylori* eradication.

The primary aim of this study was to determine eradication rates of susceptibility-guided therapy and empirical treatment in patients with treatment failure. Secondary outcomes were to investigate current antimicrobial resistance patterns and perform cost analysis comparing susceptibility-guided therapy with empirical treatment.

### 2.2 *H. pylori* culture and antimicrobial susceptibility testing

If a rapid urease test yielded a positive result, *H. pylori* culture would be subsequently performed. Two antral biopsies in an Eppendorf tube were ground in the broth. A sterile inoculating loop was used to streak culture on a Mueller Hinton II agar medium. After incubation at 37 °C in a microaerophilic atmosphere (5 % O_2_, 10 % CO_2_, and 10 % H_2_) for 3–5 days, colony formation of *H. pylori* is presented on the agar plate [14]. Fresh *H. pylori* colonies (<72 h of growth) at an inoculum concentration of 3 McFarland standards were used to perform the Epsilometer test (E-test). At 3–5 days after inoculation, the E-test strip with each antibiotic was examined to determine the minimum inhibitory concentrations (MICs) defined by the point of intersection between the inhibition ellipse and scale on the E-test strip [15]. According to the European Committee on Antimicrobial Susceptibility Testing (EUCAST), resistant strains were defined by MIC values of >0.125 mg/L for amoxicillin (AMX), >0.5 mg/L for clarithromycin (CLR), >8 mg/L for metronidazole (MTZ), >1 mg/L for levofloxacin (LVX), and >1 mg/L for tetracycline (TET) [16].

### 2.3 Definitions

**Refractory *H. pylori* infection or *H. pylori* treatment failure** was defined as persistent *H. pylori* infection after one or more treatment regimen(s) completion. Persistent *H. pylori* infection was diagnosed by a positive result of either one of tests as follows: rapid urease test, histopathology, or culture [8].

**Multidrug-resistant strains (MDR)** was defined as strains which develop resistance to at least one antibiotic in three or more classes of antibiotics [17].

**Triple therapy** consisted of proton pump inhibitor (PPI), amoxicillin (1000 mg bid), and clarithromycin (500 mg bid) for 14 days.

**Concomitant therapy** consisted of PPI, amoxicillin (1000 mg bid), metronidazole (500 mg bid), and clarithromycin (500 mg bid) for 10 days.

**Sequential therapy** consisted of PPI and amoxicillin (1000 mg bid) for 5 days followed by PPI, metronidazole (500 mg bid), and clarithromycin (500 mg bid) for 5 days.

**Quadruple therapy** consisted of PPI, bismuth subsalicylate (1048 mg bid), metronidazole (400 mg tid), and tetracycline (500 mg qid) for 14 days.

**Tailored therapy or susceptibility-guided therapy** was defined as an eradication regimen comprising antisecretory drug and susceptibility-guided antibiotics. Patients without AST performed were considered using empirical therapy.

**Early AST** was defined as AST performed immediately after first-line treatment failure.

**Nonadherence** was defined as taking less than 80 % of prescribed medication for *H. pylori* treatment (e.g., taking medication for <11 days of 14-day regimen) [18].

### 2.4 Statistical analysis

All data were analysed by using SPSS version 22 (SPSS Inc., Chicago, IL, USA). Chi-square test, or Fisher's exact test were performed for analysis of categorical variables, whereas Student's t-test was used for continuous variables and reported as mean ± standard deviation (SD). After using univariate analysis to identify factors related to achieving successful eradication after treatment failure, every variable with p-value <0.05 was further analysed by multivariate analysis to determine an adjusted p-value. All tests were two-sided and statistical significance was defined as p-value of <0.05.

### 2.5 Ethics statements

The Human Research Ethics Committee of Thammasat University (Medicine), Thailand (MTU-EC-IM-4-217/66) provided ethical approval for this study. The authors conducted this research according to the good clinical practice guideline, as well as the Declaration of Helsinki. All data had been fully anonymized before they were accessed. The Ethics Committee waived the requirement for informed consent because of no greater than minimal risk for study subjects. The authors had no accessibility to data that could identify individual participants during or after data collection.

## 3 Results

### 3.1 Demographic data of patients with *H. pylori* treatment failure

**Fig. 1:**
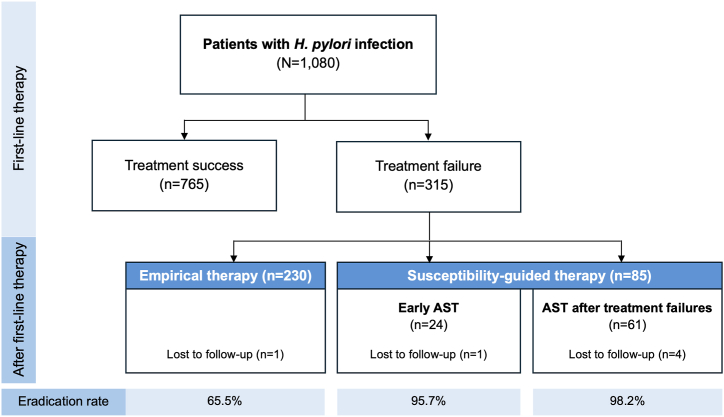
The flow diagram of patients in this study.

There were 1080 patients diagnosed with *H. pylori* infection during esophagogastroduodenoscopy at tertiary care center in Thailand. Of 1080 patients, 315 (29.2 %) had *H. pylori* eradication failure [140 men and 175 women; mean age 58.4 ± 13.1 (range 23–89) years]. Similar baseline characteristics between groups of treatment success and treatment failure were demonstrated in Table 1. The most common first-line regimen was triple therapy (40.2 %), followed by concomitant therapy (14.3 %) and bismuth quadruple therapy (14.3 %). Eradication rates of first-line regimens were as follows: triple therapy (68.1 %), concomitant therapy (74.7 %), sequential therapy (59.5 %), bismuth quadruple therapy (68.2 %), levofloxacin triple therapy (75.4 %), and vonoprazan-containing therapy (78.3 %). In multivariate analysis, sequential therapy was significantly associated with treatment failure [OR 1.66 (95%CI 1.01–2.74), p = 0.045)], whereas vonoprazan-containing therapy was associated with treatment success [OR 1.60 (95%CI 1.04–2.48), p = 0.034)]. Medication nonadherence [OR 37.97 (95%CI 8.97–160.65), p < 0.001)] contributed to treatment failure. Medication nonadherence resulted from intolerance to adverse effects such as nausea and vomiting (19/30, 63.3 %), forgetfulness (9/30, 30 %), and drug allergy (2/30, 6.7 %). Patients with treatment failure were subsequently classified into two groups. One group had no AST done and received empirical therapy (n = 230), while the other had AST done and used susceptibility-guided therapy (n = 85) as reported in Table 2. Baseline characteristics and first-line regimens between two groups were comparable. One patient in empirical therapy group and five patients in AST-guided therapy group were lost to follow-up and excluded before calculating eradication rates. The flow diagram of patients in this study was demonstrated in Fig. 1. The group of AST-guided therapy had significantly higher eradication rate than the group of empirical therapy (97.5 % vs. 65.5 %, OR 20.54; 95%CI 4.92–85.81, p < 0.001). For subgroups of AST-guided therapy, eradication rates between early AST and AST after treatment failures were not different (95.7 % vs. 98.2 %, p = 0.49). The most commonly used empirical therapy for second-line treatment was levofloxacin triple therapy (44.1 %), followed by bismuth quadruple therapy (28.8 %), and clarithromycin-containing regimens (24 %). Eradication rates of empirical treatment were as follows: levofloxacin triple therapy (69/101, 68.3 %), bismuth quadruple therapy (44/66, 66.7 %), clarithromycin-containing regimens (triple therapy 17/29, 58.6 %; concomitant therapy 12/16, 75 %; sequential therapy 3/10, 30 %). There were 8.3 % of patients in the empirical therapy group with medication nonadherence during first-line treatment. The multivariate analysis demonstrated that medication adherence was an only one factor associated with successful eradication (OR 9.84; 95%CI 1.12–86.11, p = 0.039) in empirical therapy group. All second-line regimens except for quinolone-based therapy were not different between empirical therapy group and early AST group as demonstrated in Supplementary Table 1. The proportion of quinolone-based therapy in early AST group was higher than empirical therapy group (69.6 % vs. 44.1 %, p = 0.02).

**Table 1:** Baseline characteristics of patients with treatment failure and treatment success.

Characteristics	Treatment success (N = 765)		Treatment failure (N = 315)		P-value
Male	344	(45.0 %)	140	(44.4 %)	0.875
Female	421	(55.0 %)	175	(55.6 %)	0.875
Age (years ± SD)	59.3 ± 12.9		58.4 ± 13.1		0.332
BMI (kg/m^2^ ± SD)	24.2 ± 4.1		24.4 ± 5.0		0.596
**Underlying diseases**					
None	230	(30.1 %)	91	(28.9 %)	0.701
Hypertension	286	(37.4 %)	116	(36.8 %)	0.863
Dyslipidemia	264	(34.5 %)	110	(34.9 %)	0.897
Diabetes mellitus	169	(22.1 %)	55	(17.5 %)	0.088
Cirrhosis and hepatitis	139	(18.2 %)	46	(14.6 %)	0.157
Chronic kidney disease	46	(6.0 %)	22	(7.0 %)	0.550
FH of gastric cancer	6	(0.8 %)	3	(1.0 %)	0.725
Smoking	60	(7.8 %)	23	(7.3 %)	0.761
Alcohol	109	(14.2 %)	37	(11.7 %)	0.274
**First-line treatment**					
Triple therapy	297	(38.8 %)	137	(43.5 %)	0.155
Concomitant therapy	113	(14.8 %)	41	(13.0 %)	0.453
Sequential therapy	44	(5.8 %)	30	(9.5 %)	0.026
Bismuth quadruple therapy	104	(13.6 %)	50	(15.9 %)	0.330
Quinolone-based therapy	104	(13.6 %)	34	(10.8 %)	0.210
VPZ-containing therapy	112	(14.6 %)	31	(9.8 %)	0.034
Nonadherence	2	(0.3 %)	28	(8.9 %)	<0.001
MDR strains	3/126	(2.4 %)	22/85	(25.9 %)	<0.001

BMI = body mass index, FH = family history, MDR = multidrug-resistant, SD = standard deviation, VPZ = Vonoprazan.

**Table 2:** Baseline characteristics of patients with treatment failure receiving empirical therapy or susceptibility-guided therapy.

Characteristics	Empirical therapy (N = 230)		Susceptibility-guided therapy (N = 85)		P-value
Male	109	(47.4 %)	31	(36.5 %)	0.083
Female	121	(52.6 %)	54	(63.5 %)	0.083
Age (years ± SD)	59.3 ± 13.1		56.1 ± 12.8		0.060
BMI (kg/m^2^ ± SD)	24.5 ± 5.2		24.2 ± 4.7		0.640
**Underlying diseases**					
None	61	(26.5 %)	30	(35.3 %)	0.127
Hypertension	91	(39.6 %)	25	(29.4 %)	0.097
Dyslipidemia	86	(37.4 %)	24	(28.2 %)	0.130
Diabetes mellitus	44	(19.1 %)	11	(12.9 %)	0.199
Cirrhosis and hepatitis	33	(14.3 %)	13	(15.3 %)	0.833
Chronic kidney disease	16	(7.0 %)	6	(7.1 %)	0.975
Smoking	20	(8.7 %)	3	(3.5 %)	0.118
Alcohol	29	(12.6 %)	8	(9.4 %)	0.434
**First-line treatment**					
Triple therapy	93	(40.4 %)	44	(51.8 %)	0.072
Concomitant therapy	31	(13.5 %)	10	(11.8 %)	0.688
Sequential therapy	22	(9.6 %)	8	(9.4 %)	0.967
Bismuth quadruple therapy	34	(14.8 %)	16	(18.8 %)	0.384
Quinolone-based therapy	25	(10.9 %)	9	(10.6 %)	0.943
VPZ-containing therapy	25	(10.9 %)	6	(7.1 %)	0.314
Nonadherence	22	(9.6 %)	6	(7.1 %)	0.488
**Eradication rate**	150/229	(65.5 %)	78/80	(97.5 %)	<0.001

BMI = body mass index, VPZ = Vonoprazan.

### 3.2 Antimicrobial susceptibility testing in patients with *H. pylori* treatment failure

Patients with *H. pylori* treatment failure were classified into three groups: 1) no AST (n = 230), 2) early AST (n = 24), and 3) AST after treatment failures (n = 61). AST was performed in 85 patients as demonstrated in Table 3. Most of strains (84.7 %) were resistant to antibiotics, while only 15.3 % were susceptible strains. Resistance rates to levofloxacin (LVX), metronidazole (MTZ), clarithromycin (CLR), and amoxicillin were 57.6 %, 51.8 %, 44.7 %, and 4.7 %, respectively. No tetracycline resistance was observed in this study. The group of AST after multiple treatment failures had significantly higher resistance of LVX (68.9 % vs. 29.2 %, p = 0.001), CLR (52.5 % vs. 25 %, p = 0.022), and MDR strains (32.8 % vs. 8.3 %, p = 0.020) than the early AST group. However, MTZ resistance was high in both early AST and AST after treatment failures groups, while amoxicillin resistance were low in both groups. The most common MDR organisms were CLR, LVX, and MTZ-resistant strains (21.2 %) which were only detected in the group of AST after treatment failures.

**Table 3:** Antibiotic resistance patterns of *H. pylori* strains in patients with early AST and AST after treatment failures.

Antibiotic resistance	Total patients (N = 85)		Early AST (N = 24)		AST after treatment failures (N = 61)		P-value
No resistance	13	(15.3 %)	5	(20.8 %)	8	(13.1 %)	0.504
Antibiotic resistance	72	(84.7 %)	19	(79.2 %)	53	(86.9 %)	0.504
MTZ	44	(51.8 %)	12	(50.0 %)	32	(52.5 %)	0.838
LVX	49	(57.6 %)	7	(29.2 %)	42	(68.9 %)	0.001
CLR	38	(44.7 %)	6	(25.0 %)	32	(52.5 %)	0.022
AMX	4	(4.7 %)	2	(8.3 %)	2	(3.3 %)	0.316
TET	0	(0 %)	0	(0 %)	0	(0 %)	–
**Single drug resistance**	**33**	**(38.8 %)**	**14**	**(58.3 %)**	**19**	**(31.1 %)**	**0.021**
LVX	17	(20.0 %)	4	(16.7 %)	13	(21.3 %)	0.768
MTZ	12	(14.1 %)	8	(33.3 %)	4	(6.6 %)	0.003
CLR	4	(4.7 %)	2	(8.3 %)	2	(3.3 %)	0.316
**Two drug resistance**	**17**	**(20.0 %)**	**3**	**(12.5)**	**14**	**(23.0 %)**	**0.373**
CLR and LVX	7	(8.2 %)	1	(4.2 %)	6	(9.8 %)	0.667
MTZ and LVX	5	(5.9 %)	1	(4.2 %)	4	(6.6 %)	1.000
MTZ and CLR	5	(5.9 %)	1	(4.2 %)	4	(6.6 %)	1.000
**Multidrug resistance**	**22**	**(25.9 %)**	**2**	**(8.3 %)**	**20**	**(32.8 %)**	**0.020**
CLR, MTZ, and LVX	18	(21.2 %)	0	(0 %)	18	(29.5 %)	0.003
AMX, CLR, and MTZ	2	(2.4 %)	1	(4.2 %)	1	(1.6 %)	0.487
AMX, CLR, MTZ, and LVX	2	(2.4 %)	1	(4.2 %)	1	(1.6 %)	0.487

AMX = amoxicillin, AST = antibiotic susceptibility testing, CLR = clarithromycin, LVX = levofloxacin, MTZ = metronidazole, TET = tetracycline.

### 3.3 Cost of *H. pylori* eradication in patients with treatment failure

**Fig. 2:**
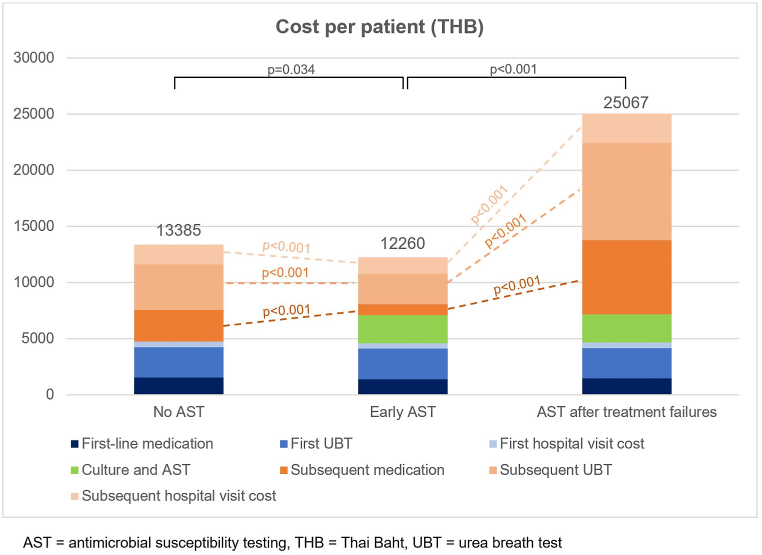
Average total cost and subcategory costs of *H. pylori* eradication per patient.

**Fig. 3:**
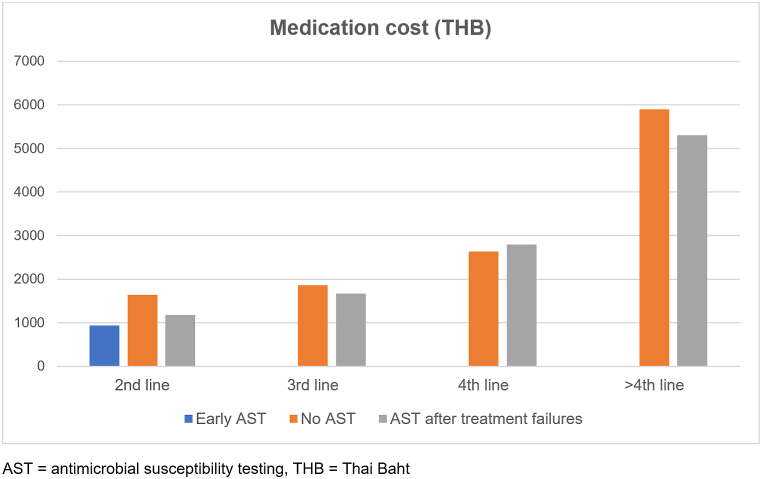
Mean medication cost after failed eradications.

Total cost of *H. pylori* eradication in patients with treatment failure was calculated as the sum of medication costs, posttreatment testing costs, hospital visit costs and AST if performed. Medication cost was calculated as the sum of cost of antisecretory drugs (PPI or potassium-competitive acid blocker) and antibiotics multiplied by duration of treatment. Urea breath test (UBT) was regularly used as posttreatment testing for refractory *H. pylori* infection. UBT cost was $81.1 [2700 Thai Baht (THB)]. The cost of *H. pylori* culture and AST was $75.1 (2500 THB). The average total cost and all subcategory costs of *H. pylori* eradication per patient were illustrated in Table 4 and Fig. 2. The mean cost of first-line medication of the group without AST, early AST, and AST after treatment failures were not different from each other ($46.8 vs. $42.6 vs. $44.1, respectively, p > 0.05). Costs of first UBT and first hospital visit were also similar among three groups resulting in comparable total cost of first-line treatment. The cost of *H. pylori* culture and AST was added in the group of early AST and AST after treatment failures. The mean cost of subsequent medication in the early AST group was significantly lower than the group without AST ($28.2 vs. $84.3, p < 0.001) and AST after treatment failures ($28.2 vs. $198.0, p < 0.001). The mean medication cost increased after every failed eradication as depicted in Fig. 3. The mean cost of subsequent UBT in the early AST group was also lower than the group without AST ($81.1 vs. $122.2, p < 0.001) and AST after treatment failures ($81.1 vs. $261.1, p < 0.001). The mean cost of subsequent hospital visits in the early AST group was also lower than the group without AST ($45.0 vs. $52.6, p < 0.001) and AST after treatment failures ($45.0 vs. $78.4, p < 0.001). Consequently, early AST group had significantly lower total average cost of *H. pylori* eradication than the group without AST ($368.2 vs. $402.0, p = 0.034) and AST after treatment failures ($368.2 vs. $752.8, p < 0.001). The early AST group had the lowest cost of subsequent medication, posttreatment UBT, and hospital visits.

**Table 4:** Average total cost and subcategory costs of *H. pylori* eradication per patient.

Cost of *H. pylori* eradication	No AST		Early AST		AST after treatment failures	
	THB	USD	THB	USD	THB	USD
First-line medication	1557	46.8	1420	42.6	1468	44.1
First UBT	2700	81.1	2700	81.1	2700	81.1
First hospital visit	500	15.0	500	15.0	500	15.0
*H. pylori* culture and AST	–	–	2500	75.1	2500	75.1
Subsequent medication	2807	84.3	940	28.2	6594	198.0
Subsequent UBT	4068	122.2	2700	81.1	8695	261.1
Subsequent hospital visits	1753	52.6	1500	45.0	2610	78.4
**Total average cost**	13,385	402.0	12,260	368.2	25,067	752.8

AST = antibiotic susceptibility testing, THB = Thai Baht, UBT = urea breath test, USD = United States Dollar.

Costs were calculated with an exchange rate of 33.3 Thai Baht to 1 United States Dollar.

## 4 Discussion

*H. pylori* eradication confers protection against gastric cancer. Nowadays, current first-line regimens provide eradication rates of less than 80 % owing to rising clarithromycin resistance [19]. Treatment failure becomes a challenging problem that needs to be solved. This study demonstrated that patients with treatment failure using susceptibility-guided therapy had significantly higher eradication rate than the group using empirical therapy. Furthermore, cost analysis also reported that the group with early AST had lower total average cost of *H. pylori* eradication than the group without AST and AST after multiple treatment failures. Early AST might be a cost-effective strategy which could enhance the eradication rate of refractory *H. pylori* infection.

Treatment failure has become a major concern in management of *H. pylori* infection in recent years. Eradication failure rate after first-line therapy in our study (29.2 %) was comparable to others (20.2%–23.4 %) [[20], [21], [22]]. Majority of patients in this study (40.2 %) and others (39.4%–80.2 %) from different global regions used clarithromycin triple therapy as first-line regimen despite high prevalence of clarithromycin resistance in all regions including Asia, Europe, and North America [[20], [21], [22]]. This could be due to discrepancy between real-world practice and guideline recommendation. Although gastroenterologists in our study followed the latest Thai guideline in 2015 which recommended triple therapy as first-line treatment [7], this study highlights that the national guideline will require revision in the near future according to declining cure rates of first-line clarithromycin-containing regimens. The reason why sequential therapy was significantly associated with treatment failure might be due to regimen complexity and high dual clarithromycin and metronidazole resistance. In contrast, vonoprazan-containing therapy as first-line regimen was a factor related to treatment success. This might be because of vonoprazan's properties as a potent acid blocker and its superior efficacy to proton pump inhibitor-based therapy for eradication of clarithromycin-resistant strains [23]. Treatment failure occurs as a consequence of either host or bacterial factors [8]. Medication nonadherence was associated with eradication failure in this study which was similar to the prior study [24]. However, it might be difficult to improve the adherence rate in this study since the adherence rate in treatment failure group was already high (91.1 %). The most common cause of medication nonadherence in this study was intolerance to side effects from antibiotics. Probiotics such as *Lactobacillus* spp and multiple strains might be beneficial for reducing antibiotic-related side effects which could further improve eradication rates [25]. Apart from patient nonadherence, antibiotic resistance is also a principal cause of treatment failure [8]. Although there were approximately 20 % of patients with AST, this study revealed higher proportion of MDR strains in treatment failure group than successful treatment group. Our important data about local antibiotic resistance patterns and previously failed regimens could assist in appropriate regimen selection after treatment failure.

Antimicrobial susceptibility testing is a valuable tool to guide antibiotic choice for refractory *H. pylori* infection. After treatment failure, this study revealed that AST-guided therapy provided significantly higher eradication rate than empirical therapy (97.5 % vs. 65.5 %). The recent meta-analysis reported no significant difference in eradication rates for rescue therapies between susceptibility-guided therapy and empirical treatment; however, there was considerable heterogeneity of studies between groups (RR 1.07; 95%CI 0.97–1.18; *I*^*2*^ 78 %) [26]. Most of empirical therapies prescribed for second-line treatment in this study were levofloxacin triple therapy and bismuth quadruple therapy yielding low cure rates (65–70 %). High level of LVX resistance (57.6 %) could explain markedly reduced second-line eradication rates. This study also demonstrated that CLR-containing regimens yielded low eradication rates (30–75 %) and should not be used as empirical therapy after treatment failure. Moreover, this study reported that resistance to LVX, CLR and MDR substantially increased after multiple treatment failures compared with failure after only one regimen. This data emphasized that repeated antibiotic exposure after each time of eradication could develop more resistant *H. pylori* strains. A similar result was observed in the previous study which demonstrated extremely high secondary resistance of CLR, MTZ, and LVX up to 50–80 %, whereas amoxicillin and tetracycline resistance remained low [27]. From AST results in our study, it can be implied that early use of AST-guided therapy not only resulted in early successful eradication, but also prevented the development of more resistant strains in the latter eradication.

Availability and the cost of AST are essential issues to take into consideration to generalize the use of AST in *H. pylori* treatment failure. This study demonstrated that early AST group had lower total average cost of eradication than the group without AST and AST after treatment failures. This was because early AST group received tailored therapy since second-line treatment and most of them achieved successful second-line eradication. Therefore, they had the lowest cost of medication after first-line treatment failure, posttreatment UBT, and hospital visits. Most of previous studies were conducted to compare cure rates of first-line treatment between tailored regimen and empirical treatment which was different from our study evaluating eradication rates after treatment failure [[10], [11], [12]]. All studies exhibited higher eradication rates of tailored treatment, while the cost was lower or comparable to empirical therapy [[10], [11], [12]]. One study also mentioned that tailored therapy not only improved the eradication rate (91.7 % vs. 82.6 %) but also achieved a cure with shorter treatment duration (79.8 vs. 99.2 days), reduced adverse events (12.9 % vs. 14.8 %), and lower total medical costs than empirical group for first-line treatment [28]. Similarly, our study demonstrated higher eradication rate and lower cost of treatment, but we highlighted this in subsequent treatment beyond first-line therapy. One previous study displayed different results from our study reporting higher cost of genotypic resistance-guided therapy ($6920 to additionally cure one patient) compared to empirical therapy but no difference in eradication rates (78 % vs. 72.2 %) [13]. This might be because of different study design and population. The study by Liou et al. was a randomized trial evaluating patients with at least 2 failed eradications, while our study assessed patients who failed first-line treatment. Furthermore, Liou et al. study demonstrated considerably higher antibiotic resistance rates (resistance to CLR 90.1–95.4 %, LVX 58.6–62.1 %, MTZ 63.5–68.2 %, AMX 13.8 %) than our study [13]. This might be implied that patients having more failed treatments would develop more resistant strains causing more difficult-to-treat refractory *H. pylori* infection [29].

Our study has several strengths. This was a large population-based study reporting better eradication rates of susceptibility-guided therapy than empirical therapy in patients with *H. pylori* treatment failure. Moreover, this research highlighted that performing early AST might be more cost-effective than AST after multiple treatment failures. However, this study also had some limitations. Firstly, there were some missing data of AST due to the nature of retrospective cohort study. We tried our best to collect as many positive culture results as possible in our analysis. Secondly, healthcare and medication costs substantially vary among countries. Moreover, AST for *H. pylori* is not available in some areas in Thailand. Therefore, this might limit generalizability in areas with different healthcare costs or lack of AST.

In conclusion, early AST is cost-effective for treatment of *H. pylori* eradication failure. Susceptibility-guided therapy provided significantly higher eradication rate than empirical therapy in patients with treatment failure. The most appropriate first-line treatment depends on local antibiotic resistance pattern. Vonoprazan-containing regimen was associated with treatment success and might be used as first-line regimen. Medication adherence should be enhanced since first-line treatment to prevent eradication failure.

## CRediT authorship contribution statement

**Natsuda Aumpan:** Writing – review & editing, Writing – original draft, Visualization, Validation, Software, Resources, Methodology, Investigation, Formal analysis, Data curation, Conceptualization. **Pornpen Gamnarai:** Writing – review & editing, Resources, Investigation. **Arti Wongcha-Um:** Writing – review & editing, Validation, Methodology, Investigation. **Muhammad Miftahussurur:** Writing – review & editing, Supervision, Methodology. **Yoshio Yamaoka:** Writing – review & editing, Validation, Supervision, Methodology, Funding acquisition. **Ratha-korn Vilaichone:** Writing – review & editing, Visualization, Validation, Supervision, Resources, Project administration, Methodology, Investigation, Funding acquisition, Formal analysis, Data curation, Conceptualization.

## Data availability statement

The supplementary data have been deposited at Figshare (https://figshare.com) with accession numbers (https://doi.org/10.6084/m9.figshare.26381728.v1).

## Ethics Declarations

This study was reviewed and approved by the Human Research Ethics Committee of Thammasat University (Medicine), Thailand, with the approval number: MTU-EC-IM-4-217/66. Informed consent was not required for this study because of no more than minimal risk to study subjects.

## Declaration of competing interest

The authors declare that they have no known competing financial interests or personal relationships that could have appeared to influence the work reported in this paper.
