# Supplementary material for: The use of real-world evidence to generate cost analysis of antibiotic susceptibility testing (AST) in patients with Helicobacter pylori treatment failure in Thailand: A large population-based study
Source: Heliyon. 2024 Oct 10;10(21):e39189. doi: 10.1016/j.heliyon.2024.e39189 (PMC11539252; doi:10.1016/j.heliyon.2024.e39189)
Supplement: Multimedia component 1 [file mmc1.docx]

**Supplementary Table 1.** Second-line regimens in the empirical therapy group and early AST group

| **Regimens** | **Empirical therapy**  **(N = 229)** | | **Early AST**  **(N = 23)** | | **P-value** |
| --- | --- | --- | --- | --- | --- |
| Quinolone-based therapy | 101 | (44.1%) | 16 | (69.6%) | 0.020 |
| Bismuth quadruple therapy | 66 | (28.8%) | 3 | (13.0%) | 0.106 |
| Triple therapy | 29 | (12.7%) | 2 | (8.7%) | 0.749 |
| Concomitant therapy | 16 | (7.0%) | 2 | (8.7%) | 0.673 |
| Sequential therapy | 10 | (4.4%) | 0 | (0%) | 0.606 |
| VPZ-containing therapy | 7 | (3.1%) | 0 | (0%) | 1.000 |
